# Effect of Meteorological Factors on *Hyalomma* Species Composition and Their Host Preference, Seasonal Prevalence and Infection Status to Crimean-Congo Haemorrhagic Fever in Iran

**Published:** 2019-09-30

**Authors:** Nayyereh Choubdar, Mohammad Ali Oshaghi, Javad Rafinejad, Mohammad Reza Pourmand, Naseh Maleki-Ravasan, Mostafa Salehi-Vaziri, Zakkyeh Telmadarraiy, Fateh Karimian, Mona Koosha, Abbas Rahimi-Foroushani, Safdar Masoomi, Kourosh Arzamani, Jalil Nejati, Mohsen Karami, Ehsan Mozaffari, Yaser Salim-Abadi, Eslam Moradi-Asl, Behrooz Taghilou, Manouchehr Shirani

**Affiliations:** 1 Department of Medical Entomology and Vector Control, School of Public Health, Tehran University of Medical Sciences, Tehran, Iran; 2 Department of Pathobiology, School of Public Health, Tehran University of Medical Sciences, Tehran, Iran; 3 Department of Parasitology, Pasteur Institute of Iran, Tehran, Iran; 4 Department of Arboviruses and Viral Hemorrhagic Fevers, Pasteur Institute of Iran, Tehran, Iran; 5 Department of Epidemiology and Biostatistics, School of Public Health, Tehran University of Medical Sciences, Tehran, Iran; 6 Vector-Borne Diseases Research Center, North Khorasan University of Medical Sciences, Bojnurd, Iran; 7 Department of Public Health, School of Public Health, Zahedan Unversity of Medical Sciences, Zahedan, Iran; 8 Department of Parasitology, Faculty of Medicine, Babol University of Medical Sciences, Babol, Iran; 9 Department of Health Services and Health Promotion, School of Public Health, Rafsanjan University of Medical Sciences, Rafsanjan, Iran; 10 Department of Public Health, School of Public Health, Ardabil University of Medical Sciences, Ardabil, Iran; 11 Zanjan Health Center, Zanjan University of Medical Sciences, Zanjan, Iran; 12 Mamasani Health Center, Shiraz University of Medical Sciences, Shiraz, Iran

**Keywords:** *Hyalomma*, Ticks, Climate, Host, Environment

## Abstract

**Background:**

The impact of environmental factors and host on *Hyalomma* spp. community structure and abundance in the main Crimean-Congo haemorrhagic fever (CCHF) foci of Iran is largely unknown.

**Methods:**

Biotic and abiotic factors, including host, temperature, humidity, altitude, Köppen-Geiger climate types, season, and precipitation on *Hyalomma* spp. community structure and abundances in 11 provinces of Iran were investigated. Additionally, the possible infection of ticks with CCHF virus was evaluated using reverse transcription PCR technique.

**Results:**

Species analyses demonstrated the presence of *Hyalomma anatolicum*, *H. marginatum*, *H. dromedarii*, *H. asiaticum*, *H. detritum* and *H. schulzei* in the study area. *Hyalomma anatolicum* was the dominant species in the southern and northern parts, whereas *H. dromedarii* was distributed mostly in central parts of the country. The highest tick infestation was recognized in hot season. Spatial variation in tick relative density was observed between habitat types where more ticks were collected in deserts, semi-deserts, and Mediterranean habitats. Except for *H. dromedarii*, which was more prevalent on camel (P= 0.044), there were no significant variations in the frequencies of other *Hyalomma* species on different hosts. *Hyalomma anatolicum*, *H. dromedarii* frequencies had significant positive and negative association with temperature and precipitation respectively. Also humidity has positive impact on *H. asiaticum* frequency.

**Conclusion:**

Data presented here will help improve ecological models to forecast the distribution of *Hyalomma* spp. ticks, to evaluate the risk of CCHF and other tick-borne diseases, and to design proper vector control measures to suppress *Hyalomma* populations in Iran.

## Introduction

Ticks are among the major vectors of pathogens for animals and humans in the world.

They can play a crucial role in the transmission of a wider range of pathogens including protozoans, viruses and bacteria than other arthropod vectors (1). The main public health problem and the largest geographical distribution related to ticks is Crimean-Congo haemorrhagic fever (CCHF), a viral hemorrhagic fever, for which ticks serve as reservoirs of the virus. Humans are infected either through tick bites or due to direct contact with infected blood and tissues of a mammalian host (2). Infected domestic animals are known to act as amplifying hosts in the absence of any clinical symptoms (3). The main mode of CCHFV transmission to humans in Iran is exposure to the blood or viscera of infected livestock. Moreover, direct exposure to ticks (tick bites) accounts for noteworthy transmission in humans (4–7). CCHF’s case mortality rate ranging from 5–80% (8, 9). CCHF virus is endemic to Africa, the Balkans and Ukraine, the Middle East and Central Asia (10). It is prevalent in most of Iran’s neighboring countries, including Pakistan and Afghanistan (11). Iran is known as one of the main foci of CCHF in western Asia where the first outbreak of the disease was reported in 1999 and has since become the main public health concern in the country (5, 12–14).

CCHF virus exists in an enzootic cycle between ticks and mammals, and geographic distribution of the virus mirrors the distribution of the primary tick vector species (15). In addition, other factors such as environment, climate, geographical features, socioeconomic parameters, grazing system, and livestock’s age can affect the distribution and transmission of the disease (16–18). Seasonal pattern, temperature, relative humidity, and lower altitude positively affect the occurrence of the disease (15). Moreover, the main foci of this disease in humans were in the eastern, northeastern, and central regions of Iran (5, 12–14). Currently, CCHF is prevalent in 23 provinces (Ps) of Iran, particularly the ones having a long border with three high-risk countries, Turkey, Afghanistan and Pakistan (9, 11, 18, 19). Since 2000, the most CCHF cases have been reported from Sistan and Baluchistan Province in southeast corner of Iran where *Hyalomma* spp. ticks are present and CCHFV is endemic (4, 15, 20).

The CCHFV has been isolated from 30 species of hard ticks; however, the main group of vectors appears to be ticks of the genus *Hyalomma* in most parts of the world (21). *Hyalomma marginatum* Koch, 1844, and *H. asiaticum* Schulze and Schlottke, 1930, are the main CCHFV vector in Europe and Asia respectively (9, 21). In Iran, the virus has been isolated from several species of hard ticks (Ixodidae) includes; *Hyalomma*, *Rhipicephalus*, *Haemaphysalis*, and *Dermacentor* genera. The most frequent species of this genus reported in Iran include; *H. marginatum*, *H. asiaticum*, *H. anatolicum* Koch, 1844, *H. detritum* Schulze, 1919, *H. dromedarii* Koch, 1844, *H. rufipes* Koch, 1844 and *H. schulzei* Olenev, 1931 (22–24).

Climate may play an essential role in the distribution and seasonal abundance of ticks (25, 26). Iran is composed of nine climate types out of 31 possible Köppen-Geiger climate types (27). Most parts of central, eastern and southern Iran are characterized by Köppen BWh (Hot desert climate) and BWk (Cold desert climate) climate types. The coastal areas of the Caspian Sea and most parts of mountainous areas of Zagros and Alborz in the west and north of Iran have moderate climate type Csa (temperate with hot and dry summer=hot summer climate). However, the eastern slope of Zagros and southern slope of Alborz connected to the central arid and semi-arid climate of central Iran are distinguished with BSk climate (Cold semi-arid climate= cold steppe). The southern parts of Zagros region are mostly dominated by BSh climate (Hot semi-arid). Dsa (Hot-summer Mediterranean) and Dsb (warm-summer Mediterranean) climate types are found in some parts of mountainous areas of Zagros and Alborz, while Csb (Warm-summer Mediterranean) and Cfa (humid subtropical) are the localized climate types found in coastal areas of the Caspian Sea. Nevertheless, very few studies on *Hyalomma* spp. community have incorporated biotic and abiotic environmental factors such as host, season, altitude, latitude, temperature, humidity, precipitation, and climate features in Iran.

In this study, we have tried to determine *Hyalomma* species community structure on livestock in different regions of Iran to assess their spatial and temporal distribution, and to test overall impact of the environmental factors on *Hyalomma* species density and diversity, and possible association with CCHF distribution in the country. We also tested their infection to CCHFV to evaluate the abundance of potential CCHFV vectors in the region. This would provide data on the relative prevalence of *Hyalomma* spp., which might facilitate the control of ticks and tick-borne diseases in the study areas.

## Materials and Methods

### Study area

**Fig. 1.**
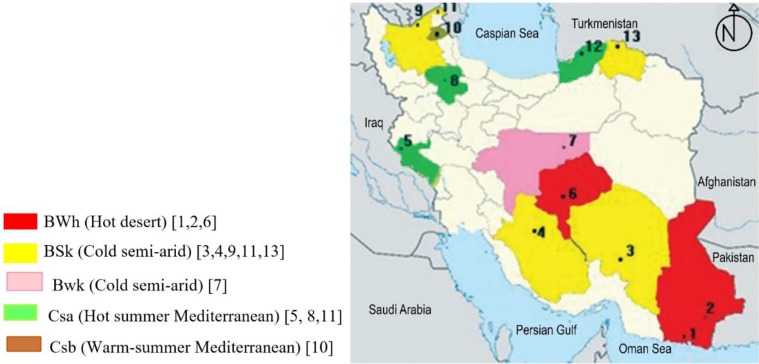
Map of Iran, tick collection sites, and climate norms of the study area. 1–2: Chabahar and Sarbaz, of Sistan and Baluchestan, 3: Raven of Kerman, 4: Marvdasht of Fars, 5: Pardeh of Ilam, 6: Mehriz of Yazd, 7: Nain of Isfahan, 8: Ijrud of Zanjan, 9: Khoda-Afarin of East Azerbaijan, 10–11: Meshgin Shahr and Pars-Abad of Ardabil, 12: Gonbad-e-Kavus of Golestan, and 13: Raz and Jargaland of North Khorasan

Iran lies between latitudes 24° and 40° N and longitudes 44° and 64° E and locates in Eurasia and Western Asia. It is bordered to the northwest by Armenia and the Republic of Azerbaijan; to the north by the Caspian Sea; to the northeast by Turkmenistan; to the east by Afghanistan and Pakistan; to the south by the Persian Gulf and Oman Sea, and to the west by Turkey and Iraq (Fig. 1). Iran is considered as an area with wide ranges of altitude (below sea-level in shores of the Caspian Sea to 5,770m of the Damavand Mountain), climate (humid and nearly jungle-like forests in the north to arid places in Dasht-e Lut with less than 100 mm annual rainfall) and temperature (from −35 °C in the northwest to 70 °C in the deserts of Dasht-e Lut).

Tick specimens were collected from eleven Ps in Iran as follows: North Khorasan, East Azerbaijan, Ardabil (Pars Abad), Kerman and Fars with BSk climate, Yazd, Sistan and Baluchistan with BWh climate, Isfahan of BWk climate, Illam, Zanjan, and Golestan of Csa climate and Meshkin Shahr of Ardabil of Csb climate (Fig. 1, Table 1).

**Table 1.** Details of meteorological variables in the study areas

**Province**	**District**	**Climate norm**	**Coordinates**	**AASL (m)**	**MAP (mm)**	**MAT (°C)**	**MARH (%)**
**Ardabil**	Meshgin shahr	Csb	38°24 ′ 50.8″ N 47°38′ 27.3″ E	1338	356	9.7	63
	Pars-abad	BSk	39°26 ′ 44.2 ″N 47°25′ 07.1″ E	32	382	12.1	63
**East Azerbaijan**	Khoda-afarin	BSk	39°08′26.5″N 46°57′27.5″E	1144	403	13.6	82
**Fars**	Marvdasht	BSk	30°01′19.7″N 52°38′22.9″E	1620	263	16.7	47
**Ilam**	Pardeh	Csa	33°46′48.2″N 46°04′26.9″E	1378	442	16.2	35
**Golestan**	Gonbad-e kavus	Csa	37°15′50.7″N 55°12′18.5″E	52	363	18.6	93
**Isfahan**	Nain	BWk	32°50′38.1″N 53°04′29.2″E	1571	76	16.6	50
**Kerman**	Rayen	BSk	29°35′00.8″N 57°28′01.2″E	1756	176	14.2	60
**North Khorasan**	Raz and Jargaland	BSk	37°56′32.4″N 57°06′16.3″E	1086	262	11.5	42
**Sistan and Baluchestan**	Chabahar	BWh	26°14′27.5″N 61°24′10.0″E	7	106	25.8	70
	Sarbaz	BWh	26°37′35.5″N 61°15′42.9″E	915	134	23.5	70
**Yazd**	Mehriz	BWh	31°35′33.7″N 54°30′25.3″E	1230	55	18.9	32
**Zanjan**	Ijrud	Csa	36°06′00.2″N 48°35′54.4″E	2050	356	10.2	53

AASL: altitude above sea level, MAP: mean annual precipitation, MAT: mean annual temperature, MARH: mean annual relative humidity, BWh: Hot desert climate, BWk: Cold desert climate, Csa: Hot summer Mediterranean climate, Csb: Warm-summer Mediterranean climate, BSk: Cold semi-arid climate

### Tick Collection

Samplings were carried out in both hot and cold seasons in 2016 and 2017. Since infected livestock is the main mode of CCHFV transmission to humans in Iran, ticks were collected from livestock during three to five visits in per season from eight farms/stables in each district. Thirteen districts were tested for the presence of ticks (Table 1). The cattle were randomly selected from farms/stables (nomadic grazing system) and whole body of 20 animals (cow, sheep, goat and or camel, based on availability) were carefully examined, just after whipping, the ticks were manually collected. Ticks were carefully removed from animals by forceps. Details of sample collection including location, type of animal, and date were recorded. Ticks were stored in tubes and covered on top with small piece of muslin cloth and carefully labeled at the collection site. All of the collected specimens were identified to species level by using a stereomicroscope (Olympus SZ51-Japanese) and a taxonomic key (28).

### Environmental factors

Environmental data were obtained from various data providers (Table 1). Elevation (altitude) and latitude/longitude data were supplied by www.gps-coordinates.org. Mean annual precipitation, relative humidity, temperature, and climate data were obtained from http://www.irimo.ir. The human CCHF cases were provided by Ministry of Health and Medical Education of Iran.

### RNA extraction and RT-PCR

Alive tick specimens were separately washed with Phosphate Buffered Saline (PBS) and crushed with pestle in 200–300μl of PBS. Total RNA was extracted from each sample homogenate using the QIAamp RNA Mini kit (Qiagen, Germany) according to the manufacturer’s instructions. The extracted RNA was dissolved in 50μl of RNase-free water and stored at −70 °C until analysis. Reverse transcription of RNA was performed individually using one-step RT-PCR kit and specific primers F2 (5′- TGGACACCTTCACAAACTC -3′) and R3 (5′- GACAAATTCCCTGCACCA -3′) (29), which amplify a 536bp fragment of the S segment of the CCHF viral genome. The total CCHFV genome, extracted from a previously confirmed RT-PCR-positive serum was used as the positive control, and no template control (NTC) was used as the negative control in the RT-PCR tests.

For gel-based analysis, 5μl of the PCR products were mixed with 1μl loading buffer and electrophoresis was performed on 2% Agarose gel and visualized on a UV Transilluminator.

### Statistical analysis

The numbers of ticks per season in each district were counted. Data for each individual *Hyalomma* tick were used to determine its species, and its association with factors such as season, host, and location. Associations between each specific tick species and categorical parameters were analyzed using χ^2^ test. Because the data were not normally distributed, Mann-Whitney and Kruskal-Wallis tests were used to assess the statistical significance of the variables. Spearman’s correlation coefficient and univariate and multivariate regression analysis were used to find possible associations between tick density and environmental factors using SPSS software (ver. 24, Chicago, IL, USA).

For statistical data analysis, we used Poisson regression analysis to test if there is an association between climate factors such as humidity, precipitation, altitude, and temperature as predictors and count of ticks and dependent variable. Differences in tick species proportions were considered as statistically significant if P< 0.05. All graphs and statistics were performed using GraphPad Prism ver. 5.00 for Windows (GraphPad Software).

## Results

### Tick composition

Overall, 3929 hard ticks were collected from the study areas including; 76 nymphs, 451 non-*Hyalomma*, and 3402 *Hyalomma* ticks. The non-*Hyalomma* ticks comprised specimens of *Rhipicephalus* (n= 274, 54.76%), *Dermacentor* (n= 168, 37.26%), *Haemaphysalis* (n= 11, 2.44%), *Boophilus* (n= 17, 3.77%), and *Ixodes* (n=8, 1.77 %) genera.

Two season investigations resulted in collection of hard ticks of the genus *Hyalomma* from different infested cattle in the study area. Details of the collected *Hyalomma* spp. tick specimens are shown in Table 2. From Mar 2016 to Dec 2017, a total of 1536 cattle from 128 farms from 13 districts were inspected and 3402 *Hyalomma* spp. ticks were collected. More than half (53.8%) of specimens were females. The cattle infestation level ranged from zero to 20 *Hyalomma* spp. ticks per animal. The number of *Hyalomma* spp. ticks in hot season were higher than cold season (58.3 vs. 41.7 %), however, the distribution of *Hyalomma* spp. was the same for two seasons (Mann-Whitney U= 57.5, P= 0.1091). *Hyalomma* spp tick infestation was observed in all study areas of Iran.

**Table 2.** Details of the collected ticks from 11 provinces of Iran

**District, Province (Climate)**	**Cold season**					**Hot season**				

	**Species**	**F**	**M**	**Total**	**Host**	**Species**	**F**	**M**	**Total**	**Host**
**Chabahar, Sistan and Baluchestan (BWh)**	*H. anatolicum*	182	11	312	Goat Cow	*H. asiaticum*	45	115	484	Goat Cow Camel
	*H. asiaticum*	23	19			*H. dromedarii*	0	150		
	*H. marginatum*	31	46			*H. detritum*	0	10		
						*H. marginatum*	7	70		
						*H. anatolicum*	11	76		
**Sarbaz, Sistan and Baluchestan (BWh)**	*H. anatolicum*	32	16	194	Goat Cow	*H. dromedarii*	76	80	407	Goat Cow Camel
	*H. marginatum*	18	13			*H. detritum*	0	4		
	*H. dromedarii*	46	52			*H. marginatum*	34	14		
	*H. schulzei*	10	7			*H. asiaticum*	16	8		
						*H. anatolicum*	132	43		
**Rayen, Kerman (BSk)**	*H. dromedarii*	32	9	72	Camel	*H. marginatum*	44	0	69	Camel
	*H. asiaticum*	0	31			*H. anatolicum*	13	0		
						*H. dromedarii*	0	12		
**Marvdasht, Fars (BSk) Pardeh, Illam (Csa)**	*H. marginatum*	8	1	9	Goat Sheep Cow	*H. marginatum*	51	8	59	Goat Goat Sheep
	*H. anatolicum*	39	0	65		*H. anatolicum*	54	0	91	
	*H. dromedarii*	8	4			*H. dromedarii*	13	6		
	*H. asiaticum*	8	0			*H. asiaticum*	11	0		
	*H. schulzei*	0	6			*H. schulzei*	0	7		
**Mehriz, Yazd (BWh)**	*H. dromedarii*	47	0	69	Goat	*H. dromedarii*	103	0	103	Camel
		12			Camel					
**Nain, Isfahan (BWk)**	*H. dromedarii*	48	8	56	Camel	*H. dromedarii*	62	53	271	Camel Cow
						*H. detritum*	0	13		
						*H. marginatum*	0	11		
						*H. asiaticum*	14	40		
						*H. anatolicum*	31	47		
**Ijrud, Zanjan (Csa)**		102	53	165	Cow Sheep	*H. anatolicum*	69	31	200	Cow
	*H. asiaticum*	0	7			*H. detritum*	0	27		
	*H. anatolicum*	3	0			*H. marginatum*	22	51		
	*H. dromedarii*									
**Khoda-afarin, East Azerbaijan (BSk)**	*H. anatolicum*	24	7	62	Cow Goat	*H. anatolicum*	98	25	241	Cow
	*H. asiaticum*	19	7			*H. asiaticum*	74	27		
	*H. dromedarii*	2	3			*H. dromedarii*	7	10		
**Meshgin-shahr, Ardabil (Csb)**	*H. marginatum*	15	42	66	Cow	*H. marginatum*	24	65	96	Cow Sheep
	*H. asiaticum*	0	9			*H. asiaticum*	0	7		
**Pars Abad, Ardabil(BSk)**	*H. marginatum*	24	17	60	Cow Goat	*H. marginatum*	15	11	39	Cow
	*H. anatolicum*	0	19			*H. anatolicum*	0	13		
**Gonbad-e kavus, Golestan(Csa)**	*H. dromedarii*	0	7	12	Camel	*H. anatolicum*	9	6	26	Sheep
	*H. marginatum*	0	5			*H. dromedarii*	0	7		
						*H. marginatum*	0	4		
**Raz and Jargaland, North Khorasan (BSk)**		46	23	78	Sheep Cow	*H. schulzei*	28	7	96	Sheep Goat
	*H. anatolicum*	0	9			*H. anatolicum*	13	9		
	*H. dromedarii*					*H. dromedarii*	5	0		
						*H. marginatum*	0	26		
						*H. asiaticum*	0	8		
**Total**		911	509	1220			959	1023	2128	

F: female, M: male, BWh: Hot desert climate, BWk: Cold desert climate, Csa: Hot summer Mediterranean climate, Csb: Warm-summer Mediterranean climate, BSk: Cold semi-arid climate

Six species of genus *Hyalomma* includes; *H. anatolicum*, *H. marginatum*, *H. dromedarii*, *H. asiaticum*, *H. detritum*, and *H. schulzei* were found to infest the animals in the study area. *Hyalomma anatolicum* and *H. detritum* with 31.9% and 1.59% were the most and the least prevalence species in the study area. The distribution of the six tick species among the provinces were not similar (Kruskal-Wallis test: P= 0.006, Kruskal-Wallis static: 16.33). Sistan and Baluchistan with six and Yazd and Fars with one *Hyalomma* species showed the most and the least diversity among eleven P’s (Table 1). Except for *H. detritum* found only in hot season, all the other *Hyalomma* species were collected throughout the year (Table 1). When the distributions of six species were compared in pairs, results showed that distribution of *H. detritum* and *H. schulzei* in the collection sites were significantly different (Mann-Whitney, U= 18.5 and 28, P= 0.0042 and 0.0247 respectively).

### Tick species found in different host species

**Fig. 2.**
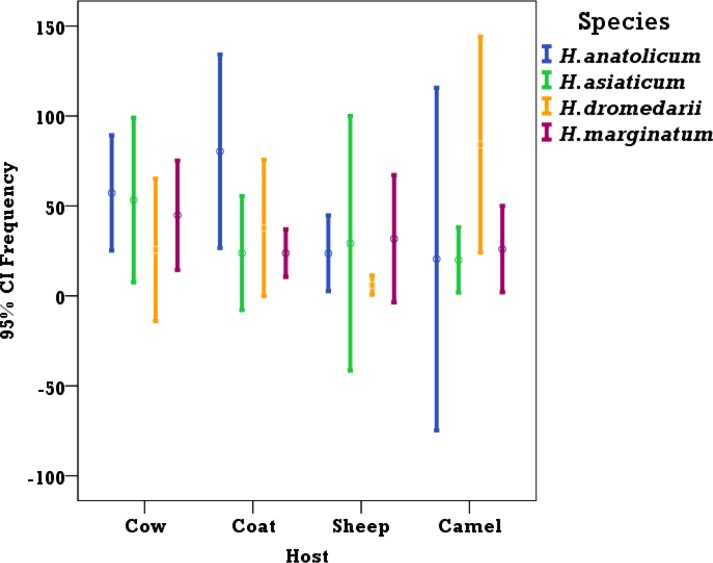
Tick species and their hosts from which they were collected. *H. dromedarii* was significantly more prevalent on camel (P= 0.044), but there were no significant variations in the frequencies of other *Hyalomma* spp on different hosts

Data were analyzed for the suitability and the specificity of the tick species to the hosts from which they were collected. Statistical analysis showed that except for *H. dromedarii*, which was significantly more prevalent on camel (P= 0.044), there were no significant variations in the frequencies of other *Hyalomma* spp on different hosts (Fig. 2). *Hyalomma anatolicum* the major tick species were found on camel, cow, sheep and goat but the number of ticks were not significantly different between the hosts (χ^2^= 4.623, df= 3, P= 0.202). *Hyalomma dromedarii* being the second most prevalent species on the hosts was significantly more abundant on camel (χ^2^= 8.104, df= 3, P= 0.044). The frequencies of *H. marginatum* and *H. asiaticum* ticks were not significantly varied (*H. marginatum*: χ^2^ = 2.008, df= 3, P= 0.544, *H. asiaticum*: χ^2^= 3.209, df= 3, P= 0.361) between the hosts, although both species were more established on cow. *Hyalomma schulzei* and *H. detritum* were rarely found and were almost exclusive on cow and cow/camel respectively.

### Impact of latitude/longitude on *Hyalomma* distribution

**Fig. 3.**
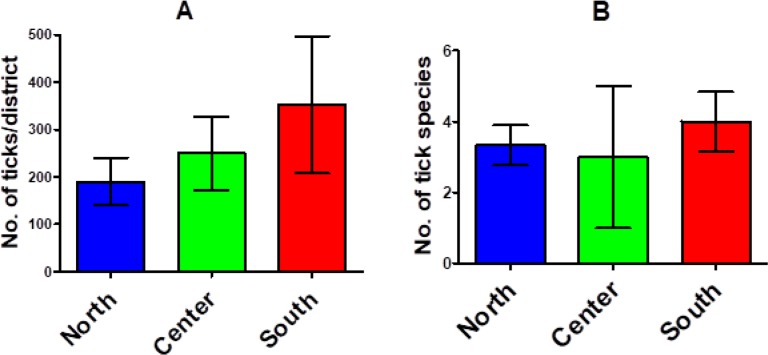
*Hyalomma* abundance (A) and species numbers (B) among northern (n= 6), central (n= 2), and southern (n= 5) districts of Iran, 2016–2017. Bars indicate mean ±SEM

To assess the impact of geographical location on *Hyalomma* species occurrence and community, the country was divided into three divisions of south (5 districts), center (2 districts), and north (6 districts). This analysis showed however, the differences were not significant (P> 0.05) among different parts of the country (Fig. 3).

### Impact of season on *Hyalomma* species abundance and community

**Fig. 4.**
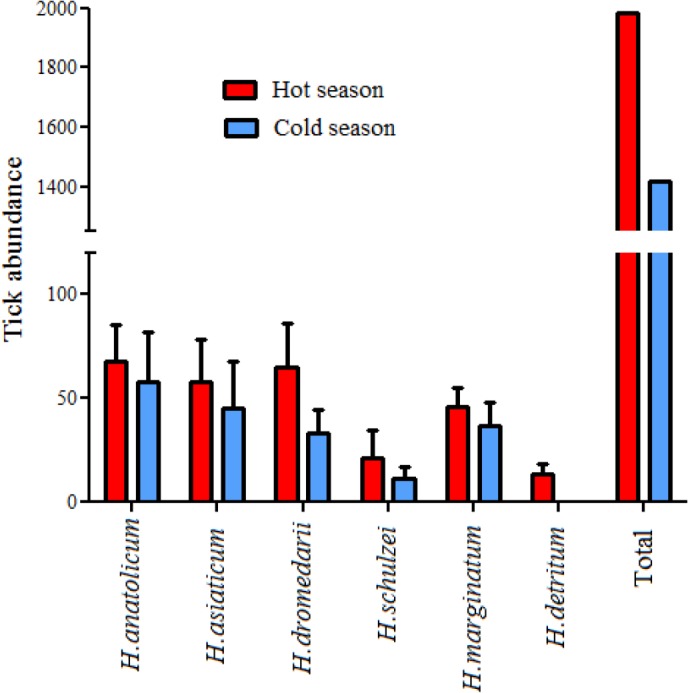
*Hyalomma* species abundances in hot and cold seasons in 13 districts of 11 provinces of Iran, 2016–2017. Bars indicate mean ±SEM, (P< 0.05). Tick abundances were significantly different in hot season for three species of *H. dromedarii*, *H. marginatum* and *H. detritum* (P< 0.05)

Seasonal analysis of tick abundances showed that seasonality positively affects *Hyalomma* spp tick abundances in the study area. Tick abundances in hot season were 1.4 times of cold season. When *Hyalomma* species were analyzed individually, tick abundances’ for all species were higher in hot season than cold season, however, differences were significant only for three species of *H. dromedarii*, *H. marginatum* and *H. detritum* (P< 0.05) (Fig. 4). Except for one location, *Hyalomma* species communities were higher in hot season than cold season (Table 1).

### Impact of temperature on *Hyalomma* species abundance and community

**Fig. 5.**
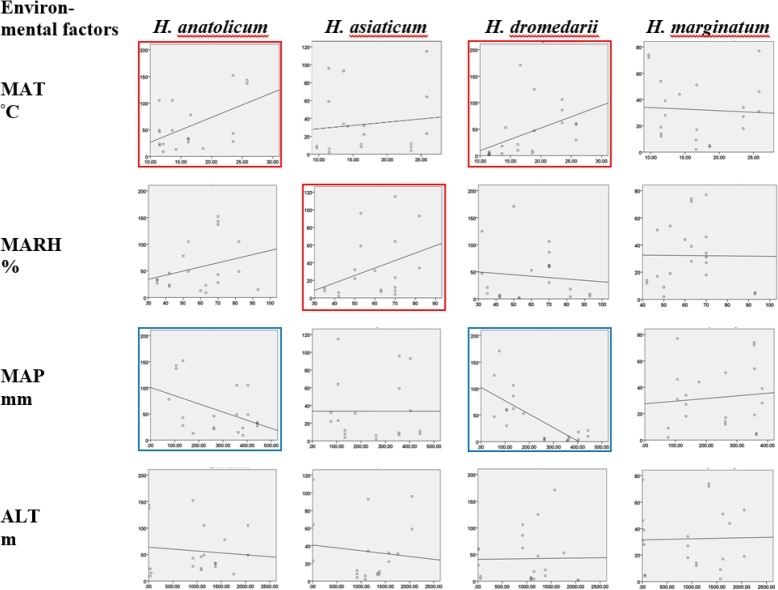
Association between environmental factors and *Hyalomma* abundances in Iran. Red squares refer to significant relationship between environmental factors and the tick species. MAT: mean annual temperature, MARH: mean annual relative humidity, MAP: mean annual precipitation, ALT: altitude above sea level

Analysis of data showed a positive association between the annual mean temperature and total tick abundances (Fig. 5). When *Hyalomma* species were analyzed individually, tick abundances’ were significantly higher for two species of *H. dromedarii* (P= 0.03) and *H. anatolicum* (P= 0.018) (Table 3).

**Table 3.** Univariate regression analysis between environmental parameters and abundance of each tick species in Iran. Bold-red represents significant correlations

** Species **	** Climate parameter **	** R Square **	** Sig **	** Parameter Constant **	** Estimates b1 **
***H. anatolicum***	Humidity	0.082	0.222	11.125	0.772
	Temperature	0.274	0.018	−20.157	4.671
	Precipitation	0.197	0.050	100.784	−0.157
	Altitude	0.011	0.667	63.813	−0.007
***H. asiaticum***	Humidity	0.122	0.143	−16.804	0.845
	Temperature	0.015	0.619	21.216	0.727
	Precipitation	0.000	0.999	33.496	−9.155E-5
	Altitude	0.013	0.647	40.882	−0.006
***H. dromedarii***	Humidity	0.012	0.641	57.447	−0.259
	Temperature	0.225	0.030	−33.177	4.264
	Precipitation	0.549	< 0.001	102.277	−0.253
	Altitude	0.000	0.942	40.866	0.001
***H. marginatum***	Humidity	0.000	0.968	33.180	−0.015
	Temperature	0.003	0.801	36.294	−0.240
	Precipitation	0.010	0.667	27.467	0.020
	Altitude	0.001	0.916	31.504	0.001

### Impact of altitude on *Hyalomma* species abundance and community

Analysis of the data revealed a weak negative association between the altitude (meter above sea level) and tick abundances (Fig. 5). Chah-bahar District in southeastern part of the country with the lowest altitude comprised the highest *Hyalomma* spp tick abundance and species community (Table 1). When *Hyalomma* species were analyzed individually, tick abundances’ differences were not significant (P= 0.647–0.947) for the species studied (Table 3).

### Impact of relative humidity on *Hyalomma* species distribution and community

Analysis of data showed there is a general positive association between the annual mean relative humidity (RH) and either the tick abundances (Fig. 5) or the tick species community. However, when each *Hyalomma* species were analyzed, tick abundances’ differences were not significant (P= 0.143–0.968) for the species studied (Table 3) suggesting no or, at best, a very weakly significant association between RH and *Hyalomma* spp present.

### Impact of precipitation on *Hyalomma* species abundance and community

On general, analysis of data showed there is a negative association between the annual mean precipitation and either the tick abundances (Fig. 5) or the tick species community. Reduction in precipitation is favored to *Hyalomma* spp tick abundance and community. However, when individual *Hyalomma* species were analyzed, tick abundances’ differences were significant only for *H. dromedarii* (P< 0.001) and *H. anatolicum* (P= 0.05) (Table 3).

### Impact of climate type on *Hyalomma* species abundance and community

**Fig. 6.**
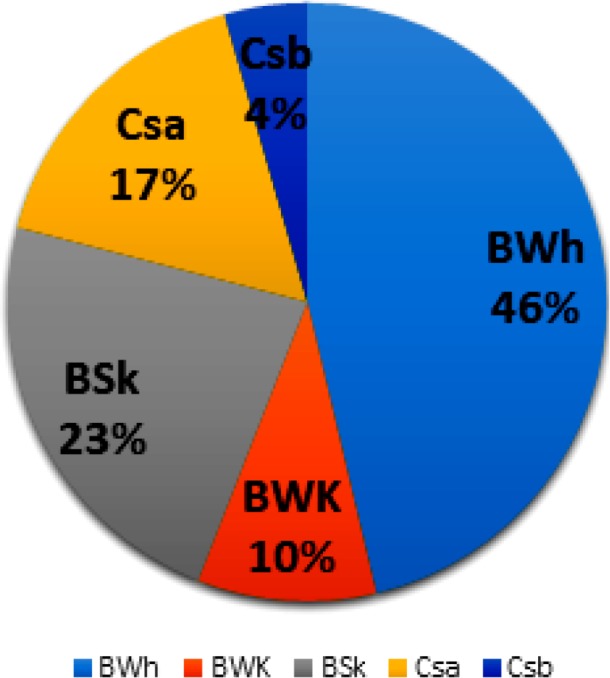
Relative abundance of *Hyalomma* ticks in five different climate norms of Iran. BSk: North Khorasan, East Azerbaijan, Ardabil (Pars Abad region), Kerman and Fars, BWh: Yazd and Sistan and Baluchistan, BWk: Isfahan, Csa: Illam, Zanjan, and Golestan, and Csb: Ardabil P (Meshkin Shahr region)

Analysis of data revealed that 46% of *Hyalomma* spp. tick specimens were collected from desert climate (BWh) in Yazd and Sistan and Baluchistan. Total tick abundance rates were significantly much lower in other climate types of the country including warm-summer Mediterranean climate (Csb) in Meshkin Shahr of Ardabil, hot-summer Mediterranean climate (Csa) in Illam, Zanjan, and Golestan, cold desert climate (BWk) in Isfahan, cold semi-arid climate (BSk) in North Khorasan, East Azerbaijan, Ardabil (Pars Abad), Kerman and Fars (Fig. 6). Except for Csb climate with two *Hyalomma* species, other climate norms had similar number (5) of *Hyalomma* species. Statistical analysis showed that abundances’ frequency of four more prevalent *Hyalomma* species was not significantly different in various climate types (P= 0.392).

### CCHFV infection

RT-PCR was used to detect the CCHF viral infection in less than 10% (390 out of 3929 ticks) of the *Hyalomma* spp ticks collected in the study areas. Among this sample (390), including all of the six *Hyalomma* species, no CCHFV positive sample was found.

## Discussion

This study is one of the first that documents the spatial-temporal as well as impact of environmental factors on abundance and community of *Hyalomma* spp. ticks in eleven provinces of Iran that include hot spots of CCHFV infection. The present study revealed the presence of six species of genus *Hyalomma* in the study area. *H. anatolicum* was the dominant species in the southern and northern parts while *H. dromedarii* distributed mostly in the central parts of the country. The genus of *Hyalomma* has known the most important tick species associated with livestock in Iran (22). Due to the importance of ticks as vector, several studies on the distribution and fauna of ticks have been performed in the country. Findings of most previous studies (30–36) are in accordance with the tick fauna and distribution reported in the present study. In the present study we have found no specimen of *H. rufipes*, *H. aegyptium*, *H. excavatum*, *H. impeltatum* which may be reflections of the sample collection method used, time and place of sampling, and ecological changes occurred in the study areas during decades.

Our results pointed out that *Hyalomma* spp. abundance in hot season was higher than cold season. This observation may be due to other or a combination of factors: greater tick activity under higher temperatures in summer, since adult *Hyalomma* spp. ticks are highly motile in seeking out vertebrate hosts (questing); human hosts are more likely to spend time outdoors during summer (longer daylight hours); increased agriculture and natural grazing for livestock during summer months leading to increased opportunity for bringing humans into contact with ticks. These factors may explain the higher incidence of the disease in the summertime in the country as stated by other researchers (12). These authors analyzed monthly CCHF passive data for 15 years (from 2000 to 2014) at the county level and showed that almost 70 % cases occurred in hot seasons (May–Sep) peaked in June with more than 180 patients out of 1027 cases (17.5%).

Our results also pointed out that temperature influences generally on *Hyalomma* spp. tick abundance where this impact was significant for *H. dromedarii* and *H. anatolicum,* the two most prevalent species in the study areas. Increasing tick vector populations directly increase the risk of disease transmission to human and hence tick abundance followed by rising temperature can be considered as an important predictor for CCHF incidence. This finding is in agreement with the epidemiological evidence indicating a significant positive correlation with CCHF disease and temperature in Iran (12, 13, 37) and other countries such as Turkey, Bulgaria (38, 39).

In the present study, we found a positive but not or weakly significant association between tick abundance and the annual mean relative humidity. This weather variable had significant positive impact on the incidence of CCHF in Iran (13, 40).

Our results also indicated that average annual precipitations affect negatively tick abundances of the two most prevalent species *H. dromedarii* and *H. anatolicum* in the study areas. These variables had reverse impact on the incidence of CCHF in Iran (14, 37, 40) and Senegal (41) Higher rainfall provide unfavorable conditions for *Hyalomma* spp. tick activity and development (42).

Results of this study also pointed out that climate norm may have impact on *Hyalomma* genus tick abundance in the study areas of Iran. Although frequencies of each species in various climate types were not significantly different, the most relative prevalence (47%) of the *Hyalomma* spp. ticks were observed in hot desert climate (BWh) comprising Yazd and Sistan and Baluchistan in southeastern and south-center of the country. The combination of economic and ecological situation, host availability, and some climate variables including duration of hot season and higher annual average temperature may support *Hyalomma* genus tick abundance and species diversity in these regions. In Sistan and Baluchistan, livestock is moved across borders and ticks could originate from distant regions. As a matter of fact, thousands of livestock (sheep, goat, and camels) are annually imported from Afghanistan and Pakistan to this province, where adult ticks carried on imported livestock from the neighboring countries is the main introductory route of CCHFV into the province (37, 43). In addition to imported livestock, migratory birds can also play an important role in *Hyalomma* spp. tick abundance and species diversity in these regions. We should consider spread of CCHFV by ticks attached to migratory birds flying thousands of kilometers during their annual migration from northern to southern areas to the province. The ecosystem of Oman Sea in Chabahar and Konarak coast in south and Hamoun-e Puzak and Helmand river in northeast of Sistan and Baluchistan, are habitats of unique species of waterfowl and shore birds as well as migratory birds (44, 45). The infected nymphs of the genus *Hyalomma* carried on migratory birds will molt on arrival into adults and then potentially infect their mammal hosts with CCHFV (46–49). However, the role of migratory birds carrying infected ticks has not been studied as a cause for increased CCHF in Iran particularly in southeast corner of Iran. It is suggested to investigate the role of the migratory birds in spreading CCHFV through attached ticks. The socioeconomic, environmental, and ecological features of Sistan and Baluchistan caused the province is being the most CCHF endemic region of the country since 2000 (4, 12, 20).

We have not found CCHF virus infection in the tick specimens collected in this study. One possible explanation for lack of CCHF infection is that although the tick specimens were collected from the animals bred on nomadic grazing system, they are usually bred intensively, and grazing is mostly limited with the field around the stables. Therefore, there are limited interactions with wildlife and their ticks, which lead to a lower tick density and diversity. Therefore, it is necessary to test effect of real nomadic grazing on the tick density and diversity in the country. Rate of animal infection to the virus was much lower (4.5%) in the animals feeding in the stable grazing system than the ones feeding in the nomadic system (30%) (17). This situation may alter abundance and community of tick population as well. Other possible explanation could be the lower age of animals we tested, where it was shown that rate of infection increased in older animals from 4.5% to 23.7% (17). However, the epidemiology of CCHF virus in Iran reviewed and *Rhipicephalus sanguineus*, *H. marginatum*, *H. anatolicum*, *H. asiaticum* and *H. dromedarii* were the most frequent species which were positive for CCHF virus (50). However, the infection rate was highly variable and ranged 0–16.7% for the tested tick species (48, 50, 51). In this study we had focused on *Hyalomma* species and did not test the virus infection in other tick species such as *Rhipicephalus* and *Haemaphysalis* which also play a role in the cycle of transmission of the disease in nature in the wild cycle and in connection with humans in the domestic cycle and should be considered in CCHF epidemiological studies (48, 50, 51).

Further studies should consider other factors influencing tick life cycle and abundance over long time, including microhabitats, nomadic (natural) system, and host density, three potentially influent factors that were not assessed in the present study. Our study was performed in one year, but these climate factors should be assessed for several years.

## Conclusion

*Hyalomma* spp. ticks are responsive to changes in their environment and may be particularly sensitive to host, season, temperature, relative humidity, and precipitation, considered as important factors of tick population dynamics. Further studies assessing the impact of microclimates and host abundance in *Hyalomma* spp. abundance and species would support our understanding of the environmental factors influencing tick prevalence and diversity.
